# Dyslipidaemia in HIV-infected women on antiretroviral therapy. Analysis of 922 patients from the Spanish VACH cohort

**DOI:** 10.1186/1472-6874-11-36

**Published:** 2011-08-04

**Authors:** Vicente Estrada, Paloma Geijo, Manuel Fuentes-Ferrer, María Luisa García Alcalde, María Rodrigo, María José Galindo, Agustín Muñoz, Pere Domingo, Esteve Ribera, Jaime Cosín, Pompeyo Viciana, Fernando Lozano, Alberto Terrón, Antonio Vergara, Ramón Teira, Josefa Muñoz-Sánchez, Bernardino Roca, Trinitario Sánchez, José López-Aldeguer, Elisabeth Deig, Francisco Vidal, Enric Pedrol, Manuel Castaño-Carracedo, Teresa Puig, Myriam Garrido, Ignacio Suárez-Lozano

**Affiliations:** 1 Hospital Clínico San Carlos, Madrid, Spain; 2 Hospital Virgen de la Luz, Cuenca, Spain; 3 Hospital Clínico San Carlos, Madrid, Spain; 4 Hospital de Cabueñes, Asturias, Spain; 5 Hospital Clínico San Carlos, Madrid, Spain; 6 Hospital Clínico, Valencia, Spain; 7 Hospital Infanta Cristina, Badajoz, Spain; 8 Hospital Santa Creu i S. Pau, Barcelona, Spain; 9 Hospital Vall d'Hebron, Barcelona, Spain; 10 Hospital Gregorio Marañón, Madrid, Spain; 11 Hospital Virgen del Rocío, Sevilla, Spain; 12 Hospital de Valme, Sevilla, Spain; 13 Hospital SAS, Jerez, Spain; 14 Hospital Clínico Puerto Real, Spain; 15 Hospital Sierrallana, Torrelavega, Spain; 16 Hospital Basurto, Bilbao, Spain; 17 Hospital General, Castellón, Spain; 18 Hospital Virgen del Rosell, Cartagena, Spain; 19 Hospital La Fe, Valencia, Spain; 20 Hospital General, Granollers, Spain; 21 Hospital Joan XXIII, Tarragona, Spain; 22 Xarxa Social i Sanitaria Santa Tecla, Tarragona, Spain; 23 Hospital Carlos Haya, Málaga, Spain; 24 Hospital Arnau Vilanova, Lleida, Spain; 25 AM-VACH, Huelva, Spain; 26 Hospital Infanta Elena, Huelva, Spain

## Abstract

**Background:**

Information concerning lipid disturbances in HIV-infected women on antiretroviral therapy (ART) is scarce. The objective of the study is to describe the lipid profile in a large cohort of HIV-infected women on contemporary ART and analyse differences between regimes and patient's characteristics.

**Methods:**

Observational, multicentre, cross-sectional study from the Spanish VACH Cohort. 922 women on stable ART without lipid-lowering treatment were included.

**Results:**

Median age was 42 years, median CD4 lymphocyte count was 544 cells/mm3, and 85.6% presented undetectable HIV-1 viral load. Median total cholesterol (TC) was 189 mg/dL (interquartile range, IQR, 165-221), HDL cholesterol 53 mg/dL (IQR, 44-64), LDL cholesterol 108 mg/dL (IQR, 86-134), and triglycerides 116 mg/dL (IQR, 85-163). Mean accumulated time on ART was 116 months; 47.4% were on NNRTI-based regimes, 44.7% on PI, and 6.7% on only-NRTI therapy. 43.8% were also hepatitis C (HCV) coinfected. Patients on PI treatment presented higher TC/HDL ratio than those on NNRTI (p < 0.001). Significantly higher HDL values were observed in NNRTI-treated patients. HCV-coinfected patients presented lower TC/HDL ratio than the non HCV-coinfected. In multivariate analysis, factors independently associated with TC/HDL ratio were age, triglyceride levels and HCV co-infection. PI treatment presented a non-significant association with higher TC/HDL ratio.

**Conclusions:**

In HIV-infected women, the NNRTI-based ART is associated with a better lipid profile than the PI-based. Factors unrelated to ART selection may also exert an independent, significant influence on lipids; in particular, age, and triglyceride levels are associated with an increased TC/HDL ratio while HCV co-infection is associated with a reduced TC/HDL ratio.

## Background

The increase in cardiovascular risk (CVR) observed in HIV-infected patients is a cause for concern. Most clinical studies have detected a relationship between cardiovascular disease and traditional risk factors, among which age, male gender, smoking, hypertension and diabetes are the most important. There is far less information concerning CVR in HIV-infected female patients. For years, some inequalities in female participation in clinical studies have been observed. In HIV infection, women have been under-represented as participants as trial participants in for all types of clinical interventions [1]. This is in sharp contrast with the fact that more than 50% of HIV-infected adults worldwide are women [2], and incidence figures show a significant increase in the number of newly-infected HIV infected women. Since the beginning of the epidemic, the number of women diagnosed with HIV/AIDS has risen more than 3-fold from 8% of all cases in 1985 to 27% in 2006 [3].

There is concern about the potential cardiovascular complications of antiretroviral therapy (ART) in women because of their characteristics and the limitations of published studies. Physiological ageing and also menopause increases CVR. Factors potentially involved in determining sex differences in pharmacological effects that may be involved in CVR include differences in body weight and composition, pharmacokinetic issues related to drug metabolism and other, such as nutritional factors, concomitant treatments as well as hormonal and reproductive status [4].

Lipid disturbances are frequently observed in HIV infection, and they include elevations in triglycerides (TG) and total cholesterol (TC), and reduced levels of high-density lipoprotein cholesterol (HDL). They may be related to ART and also to the direct effect of HIV. Low TG and high HDL levels have been described in HIV infected women in comparison to men [5], but HIV-specific and host factors may influence these differences. In studies on women from the general population, HDL and TG are independent predictors of CV disease-related death [6]. There are few data regarding the effect of gender on lipids in HIV-infected patients. Therefore, the main objective of our study is to describe the lipid profile in a contemporary large cohort of HIV infected women on ART and analyse differences between regimes and patient's characteristics.

## Methods

### Design of the study

This is a multicentre, cross-sectional study, designed to describe the lipid profile, CVR factors and HIV-related variables in a cohort of HIV-infected women on ART without lipid-lowering treatment

### Study Population

Patients were included in the Spanish VACH cohort. Characteristics of this cohort have been described elsewhere [7]. In summary, the VACH cohort collects clinical information of HIV-infected patients form 47 centres throughout the Spanish geography. Nearly 15,000 patients, aged 16 years or older, were included in the cohort at the time of the study. Clinical data were recorded in a common computer database (ACyH^®^, Betek 43 SL, Huelva, Spain). All patients gave their written informed consent prior to the inclusion in the study.

### Inclusion/exclusion criteria

Female subjects aged 18 years or more at the time of enrolment with documented HIV-1 infection, attending VACH cohort outpatient HIV-1 treatment centres for routine, scheduled, clinical appointments, were eligible for this study. In order to be eligible, subjects must have been on at least three antiretroviral drugs, at the time of the study visit. Only those patients with more than three months on the current treatment were included in the study, and no upper limit of treatment length was set. Being on any lipid-lowering treatment was an exclusion criterion. Antiretroviral (ARV) naïve subjects or ARV experienced, but currently untreated subjects or those currently treated with NRTI bi- or mono therapy were not eligible for this study. Informed consent was obtained from the patients at study entry data collection in the VACH Cohort.

### Data collecting and variables analysed

Data were prospectively collected according to standardised criteria and are electronically stored in the ACyH^®^, an application specifically developed for the management of the cohort data. On enrolment, standardised data collection electronic forms were completed at the sites providing information from physical examination, patient interview and patient case notes, concerning family history of coronary heart disease, patient prior history of CVD and diabetes, cigarette smoking, blood pressure, diabetes mellitus and anti-hypertensive therapy and fasting serum lipid levels. Physical examination included anthropometric determinations such as weight, height, waist and hip diameters. All laboratory measurements were obtained in at least 8 hours of fasting state at each site. Lipid variables analysed included TC, TG and HDL; analysis were determined by enzymatical methods using standardised commercial kits; LDL was calculated by Friedewald's formula, except in patients with levels of cholesterol over 400 mg/dL (LDL = TC - ([TG/5] + HDL). The main variable analysed was TC/HDL ratio. This was selected because of its proven value in predicting cardiovascular outcomes in both genders [8]. HIV-related laboratory variables include CD4 lymphocyte count and HIV-1 viral load. Renal function variables included creatinine plasma levels and estimated glomerular filtration ration rate (eGFR), which was calculated using the MDRD formula. The diagnosis of hepatitis C infection (HCV) was established in the presence of antibodies to hepatitis C virus. Information was unavailable regarding other important variables such as viral load, fibrosis degree or whether the patient had received specific treatment or not.

Additionally, all cumulative data characterising the patient's underlying HIV-1 infection since inclusion in any of the individual cohorts were collected, including information on demography, antiretroviral therapy, CD4 cell counts and HIV-1 viral load. Dates of diagnosis of all AIDS-defining diseases were recorded using the 1993 clinical definition of AIDS from the Centres for Disease Control and Prevention.

### Antiretroviral therapy

Most patients (98.8%) were on at least two nucleoside reverse transcriptase inhibitors (NRTI) therapy. 48.6% were also receiving non-nucleoside NRTI (NNRTI) and 45.9% protease inhibitors (PI). No information regarding specific drugs was available for analysis; in most cases, PI were low-dose ritonavir-boosted. Four groups of different treatments were identified: group 1, 2 NRTI + 1 PI (47.4% of the study population), group 2, 2 NRTI + 1 NNRTI (44.7%); group 3, 3 NRTI (6.7%) and group 4, NNRTI + PI combination therapy (1.2%). This information is also presented in Table 1.

**Table 1:** Patient's clinical characteristics

	n (%)
Age*	42.1 ± 8.3
CD4 lymphocyte count (cells/mm3)**	544.5 (373.5-759.5)
CD4 nadir (cells/mm3)**	184.5 (76-275)
**Undetectable HIV-1 viral load (<50 cop/mL)**	789 (85.6)
**Previous AIDS diagnosis**	241 (26.1)
**Hepatitis C (n = 812)**	369 (45.7)
**Genotype HCV (n = 198)**	
1	113 (57.1)
4	47 (23.7)
3	36 (18.2)
2	2 (1.0)
**Hepatitis B (n = 150)**	33 (4.3)
**BMI (Kg/m**^**2**^)*	23.7 (4.4)
**Current smoker**	505 (54.8)
**Hypertension**	153 (16.6)
**Diabetes**	24 (2.6)
**Way of transmission**	
Sexual	570 (61.8)
Injectable drug use	299 (32.4)
Blood derivatives	14 (1.5)
Vertical transmission	7 (0.8)
Unknown	32 (3.5)
**Previous CV disease**	13 (1.4)
**Familiar history of premature CVD**	46 (5.0)
10-year CV risk (Framingham)**	2% (1-4)
Total cholesterol (mg/dL)* (n = 907)	194.2 (±45.2)
HDL cholesterol (mg/dL)* (n = 907)	54.9 (±16.3)
CT/HDL ratio* (n = 907)	3.7 (±1.1)
LDL cholesterol (mg/dL)* (n = 903)	111.8 (36.6)
Triglycerides (mg/dL)**	116 (85-163)
**Patients with hyperlipidemia §**	480 (52.7)
Glucose (mg/dL)*	94.7 (±24.2)
Creatinine (mg/dL)*	0.78 (±0.19)
eGFR (mL/min)*	90.3 (76.9-107.2)
**Current antiretroviral therapy**	
NRTI + NNRTI (Group 2)	437 (47.4)
NRTI + PI (Group 1)	412 (44.7)
Only NRTI (Group 3)	62 (6.7)
NRTI + PI + NNRTI (Group 4)	11 (1.2)
**Thymidine NRTI**	180 (19.5)
Median time on current treatment (months)**	22 (11-36)
Accumulated time on ART (months)**	116 (71-149)

N = 922.

* Media (±standard deviation); ** Median (Interquartile range)**; BMI**: body mass index, **eGFR**, estimated glomerular filtration rate by Modification of Diet in Renal Disease (MDRD) formula. § **Hyperlipidemia**: (any of the following: TC >200, LDL > 130, TG > 200, CT/HDL ratio >4.5); **PI**: protease inhibitor; **NRTI**: nucleoside reverse transcriptase inhibitor; **NNRTI**: non-nucleoside reverse transcriptase inhibitor. ART, antiretroviral therapy.

### Statistical analysis

The quantitative variables were summarized by their mean and standard deviation (SD) or median and interquartile range (IQR: P_25_-P_75_). The t-student test was used to compare quantitative variables between two independent groups. The analysis of variance (ANOVA) or the non-parametric Kruskal-Wallis test was used to compare the metabolic parameters between the four treatment groups. If a significant difference was found in ANOVA or Kruskal-Wallis test, it was further evaluated by using a Bonferroni-corrected α level of 0.05/6 = 0.0083.

The relationship between the quantitative factors and the TC/HDL ratio was evaluated with a correlation analysis. The Pearson correlation coefficient was calculated when the two quantitative variables were normally distributed, and the non-parametric Spearman rank correlation coefficient when the variables were not normally distributed. A multiple linear regression analysis was carried out in order to evaluate the independent effect of each factor.

Null hypothesis was rejected by a type I error minor than 0.05 (α < 0.05). Statistical analyses were performed using the SPSS 15.0 statistical package.

## Results

922 patients were included in the study. Their clinical characteristics are described in Table 1. In general, the population study was composed of young, premenopausal age patients with a low-risk lipid profile and well-controlled HIV infection. Of note, was the proportion of HCV-coinfected patients being high (43.7%). In these patients, the most frequent HCV genotype was 1. The main CV risk factor was tobacco smoking in 54.8% of the study population.

**Figure 1:**
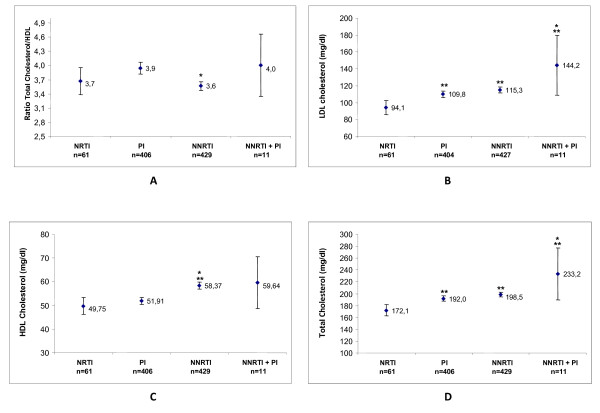
**A-D: Graphics illustrating the level of the parameters evaluated**. The data are presented as mean ± CI 95%. * Significant differences in relation to the PI treatment group; ** Significant differences in relation to the NRTI treatment group. ANOVA of one way, and after Bonferroni correction, p < 0.05; PI: protease inhibitor; NNRTI: non nucleoside reverse transcriptase inhibitor; NRTI: nucleoside reverse transcriptase inhibitor; CI: confidence interval.

The figures 1A-D shows the comparison between the different treatment groups and the metabolic parameters (total cholesterol, LDL, HDL and TC/HDL ratio). Globally, significant differences were found between the treatment groups in all the metabolic parameters (p < 0.011 for all). Regarding the TC/HDL ratio, (figure 1A) using the Bonferroni test for multiple comparisons, only statistical differences were detected between the PI and NNRTI groups (p < 0.001). LDL levels in the NRTI group were significantly lower than in PI, NNRTI and NNRTI + PI groups (p = 0.009; p < 0.001; p < 0.001, respectively) (figure 1B). Significant differences were also found between PI and NNRTI + PI groups (p = 0.011). Higher HDL values were observed in NNRTI group, and multiple comparison between groups showed significant differences between NRTI vs NNRTI (p = 0.001) and PI vs NNRTI (p < 0.001) groups (figure 1C). TC levels in the NRTI group were significantly lower than in PI, NNRTI and NNTR + PI groups (p = 0.007; p < 0.001; p < 0.001. respectively) (figure 1D). We also found significant differences between PI and NNRTI + PI groups (p = 0.0015).

Regarding TG levels, in the NRTI, PI, NNRTI and NNRTI + PI groups median values and IQR were as follows (mg/dL): 126.5 (88.5-173.2), 128.5 (94.2-183.0), 103.0 (77.0-145.7) and 141.0 (104.0-201.0), respectively. Using Kruskall-Wallis test, statistically significant differences were found between the four treatment groups (p < 0.001 for all). Using Bonferroni test for multiple comparisons, statistical differences were detected between PI and NNRTI groups (p < 0.001).

Table 2 shows mean values, SD and comparisons of TC/HDL ratio in different categories of epidemiological and clinical factors. Diabetes diagnosis and PI-treatment was associated with statistically significant higher TC/HDL ratios. There were no differences on values between patients on thymidine-NRTI vs non-thymidine NRTI. Significantly lower TC/HDL ratios were found in patients on NNRTI treatment and HCV-coinfected. No significant differences on TC/HDL ratio were found between HCV genotypes.

**Table 2:** Univariate analysis of clinical and epidemiological factors and TC/HDL ratio

	n	TC/HDL ratio (mean ± SD)	p
Previous AIDS			
Yes	230	3.85 ± 1.12	0.165
No	677	3.71 ± 1.11	
HIV-1 viral load			
Detectable	131	3.89 ± 1.20	0.099
Undetectable	776	3.72 ± 1.10	
Hypertension			
Yes	149	3.87 ± 1.26	0.156
No	758	3.72 ± 1.09	
Previous CVD			
Yes	13	3.76 ± 1.42	0.958
No	894	3.74 ± 1.12	
Diabetes			
Yes	23	4.43 ± 1.59	0.003
No	894	3.73 ± 1.10	
Type of smoker			
Current smoker	496	3.79 ± 1.18	0.193
Ex-smoker	61	3.88 ± 1.36	
Never smoker	350	3.67 ± 0.97	
T-NRTI treatment			
Yes	178	3.68 ± 1.03	0.414
No	729	3.76 ± 1.13	
PI treatment			
Yes	417	3.94 ± 1.23	<0.001
No	490	3.58 ± 0.98	
NNRTI treatment			
Yes	440	3.58 ± 0.97	<0.001
No	467	3.91 ± 1.23	
HBV coinfection			
Positive	31	3.88 ± 1.25	0.462
Negative	728	3.73 ± 1.11	
HCV coinfection			
Positive	360	3.63 ± 1.15	0.034
Negative	438	3.80 ± 1.08	

**SD**: Standard deviation; **AIDS**: Acquired immune deficiency syndrome; **Previous CVD**: previous history of cardiovascular disease: **T-NRTI**: Thymidine NRTI (AZT or d4T); **PI**: protease inhibitor; **NNRTI**: non-nucleoside reverse transcriptase inhibitor; **HBV**: hepatitis B virus; **HCV**: hepatitis C virus.

Pearson's and Spearman's correlation coefficients were calculated to evaluate the linear relationship between TC/HDL ratio and quantitative independent factors. TC/HDL ratio showed a positive significant correlation with LDL (r = 0.523; CI 95%, 0.474, 0.569, p < 0.001) and TG levels (r = 0.487; CI 95%, 0.436, 0.535, p < 0.001). Significant but weaker correlations were also found with age (r = 0.131; CI 95% 0.067, 0.194, p < 0.001) and BMI (r = 0.151; CI 95% 0.087, 0.214, p < 0.001). These positive correlations indicate that any increase in these values is associated with higher levels of TC/HDL ratio, and therefore with impaired metabolic status. To the contrary, TC/HDL ratio showed a significant negative correlation coefficient with the eGFR (r = -0.068; CI 95% -0.133,-0.002, p = 0.042).

In order to evaluate the role of the different independent factors in the TC/HDL ratio, a multiple linear regression analysis was performed. All the independent factors that showed a significant relationship with the TC/HDL ratio in the univariate analysis (age, BMI, triglycerides, eGFR, diabetes, PI treatment, NNRTI treatment and HCV coinfection) were included in the regression analyses. Of those factors, only HCV coinfection, age and TG levels remained significantly and independently associated with the TC/HDL ratio on multivariate analysis. PI-treatment and BMI reached borderline statistical significance (table 3).

**Table 3:** Multivariate analysis

	β coefficient	CI 95% β coefficient	P
Age (years)	0.009	0.001;0.017	0.045
BMI (kg/m^2^)	0.016	-0.0001;0.032	0.052
Triglycerides (mg/dl)	0.006	0.005;0.007	<0.001
eGFR	-0.002	-0.005;0.001	0.279
Diabetes	0.064	-0.345;0.473	0.757
PI treatment	0.261	0.000;0.522	0.050
NNRTI treatment	0.046	-0.214;0.306	0.728
HCV coinfection	-0.217	-0.355;-0.079	0.002

Factors independently related to TC/HDL ratio (N = 785).

BMI: body mass index; eGFR, estimated glomerular filtration rate by Modification of Diet in Renal Disease (MDRD) formula; CI: confidence interval; PI: protease inhibitor; NNRTI: non nucleoside reverse transcriptase inhibitor; HCV: hepatitis C virus.

The interaction between the PI treatment group and the TG levels with the TC/HDL ratio was studied and an interaction term was included in the linear regression model. The quantitative TG variable was dichotomized in two groups (TG > 200 mg/dl). The β coefficients, adjusted for all the variables in the model described previously, for the relationships between PI treatment and the TC/HDL ratio in the TG ≤ 200 mg/dl and TG > 200 groups were 0.21 (CI 95%: -0.08;0,49; p = 0,154) and 0.45 (CI 95%: 0.01;0,89; p = 0.044), respectively. The interaction term did not reach statistical significance (p = 0.252), showing that the relationships between variables TC/HDL ratio and PI treatment group were not modified as a function of the TG level.

## Discussion

In this study we present the lipid profile of a large cohort of HIV-infected women on contemporary ART, without lipid-lowering treatment. Most of the patients had low-risk lipid profile. The main findings include the importance of age, trygliceride levels, HCV coinfection, type of treatment (PI vs NNRTI) and BMI on plasma lipids.

Major guidelines for lipid management are based in LDL concentrations [9], although other parameters such as lipoprotein TC/HDL ratios may be better predictors of CV disease [10]. We selected TC/HDL ratio as a major marker of CVD risk and the primary objective of our analysis because of the greater predictive value than the isolated parameters [11].

Although there are no differences related to gender with regard to the efficacy of ART, an increased proportion of ART-associated adverse events have been described in women. Some drug-related complications such as lactic acidosis, anaemia, liver toxicity or nevirapine-related rash are more common in women. In the recently published darunavir/ritonavir-based Grace trial [12], which was designed to enrol a high proportion of treatment-experienced women, no sex differences in efficacy were found, but the rate of treatment discontinuation was significantly higher in women (32.8%) compared with men (23.2%). The primary reasons for study discontinuation were loss to follow up and adverse effects (AE); however, no trends toward a specific type of AE driving discontinuations in either group were found.

Information about dyslipidaemia in HIV-infected women on ART is scarce. This may be due to the reduced number of women included in clinical studies or to the fact that they have not assessed gender as an independent variable. A small observational study published in 2001 described that metabolic adverse effects during ART were more pronounced in women than in men [13]. To the contrary, Richter et al [14] described older age, white race, PI use and male sex as significant factors associated with dyslipidaemia. HIV-related fat distribution abnormalities, commonly associated with lipid disturbances, have been described in the past as more frequent in women [15]. Our results are similar to those of Anastos et al [16], who presented the largest series of patients treated women to date; they found a dyslipidaemic pattern only in HIV-infected female patients on PI-containing therapy.

It is well known that increased age is related to a pro-atherogenic lipidic profile. In fact, several cross-sectional population studies have demonstrated that TC and LDL levels increase after the onset of puberty until 50 years of age, and then plateau until 70 years of age [17]. In addition, in women HDL levels decrease with age [18], and the transition from pre-menopause to post-menopause is associated with reduction in HDL and increase in the levels of LDL, TG, Lp (a), which are independent CV risk factors [19].

Our data on the effect of HCV infection on lipid metabolism are consistent with previous studies, which describe lower lipoprotein levels (TC, LDL and HDL) in HCV mono-infected patients in comparison with control subjects [20]. HCV infection is associated with enhanced lipogenesis, reduced secretion, and beta-oxidation of lipids [21]. This described finding is more pronounced in patients infected with HCV genotype 3a [22]. In HIV/HCV coinfected patients, low TC levels have been reported, independently of the use of PI or NNRTI. In a study of high-risk for insulin resistance (IR) in hispanic men HIV/HCV co-infected patients [23], HCV mitigated the elevation of triglycerides associated with ART and modified the relationships between TG, IR and HDL. In our study population, 43% of the patients were also HCV coinfected and we found it to be an independent factor associated to lower TC/HDL ratio. However, despite a favourable lipid profile, HCV infection is associated with a higher risk of coronary artery disease after adjusting for traditional risk factors [24]. A recently published systematic review [25] has found a high likelihood of having carotid atherosclerosis in HCV-monoinfected patients. The reasons for this discrepancy are not well understood. Moreover, systemic inflammation, which plays a significant role in development and atherosclerosis progression, is not significantly different in HCV-positive and HCV-negative patients [26]. HCV viral particles have been detected in atheromatous plaques [27], suggesting HCV could play a role in carotid atherosclerosis through local action. Some HCV proteins can cause oxidative stress with increased local reactive oxygen species [28], supporting the hypothesis that HCV could potentiate the oxidation of lipoprotein. An increased oxidant stress has been described in HIV-infected women [29]. A significant association was found between levels of oxidant stress as measured by plasma F2-isoprostane concentrations and HCV viremia, transaminase level, waist circumference and homocysteine levels. Therefore, in this case a favourable lipid profile should not be construed as a protection against atherosclerosis.

It has long been known that ART has a significant effect on plasma lipids [30]. Normalization in lipid values is usually observed in naïve patients after beginning ART and in general, a rise in TC, TG and LDL is observed in patients on stable ART. A pro-atherogenic lipid profile, with increased number of small LDL particles and a reduced number of small HDL particles has been described in a large study of female HIV-patients on ART [31]. There is a wide range variation in the elevation in lipids levels in HIV infected patients that may be influenced by multiple factors [32]. In general, the use of PI/r is associated with higher elevations in TC and TG than with the use of NNRTI. There is no clear information about the influence of gender in these lipid derangements. We have shown that in HIV infected women on ART, the use of PI or NNRTI is associated with an opposite effect on TC/HDL ratio, and NNRTI use is associated with a better lipid profile. In univariant analysis, the use of NNRTI is associated with a reduced TC/HDL ratio when compared to PI. In multivariable analysis a borderline effect of PI on TC/HDL ratio is observed, suggesting a partially unrelated to ART pathogenesis of lipid changes in HIV-infected women. This beneficial effect on the lipids of the NNRTI may have clinical relevancy, provided that the antiretroviral treatment has to be kept indefinitely and considering the fact that aging associates with an increase of cardiovascular risk.

The use of thymidine-NRTI therapy (d4T or AZT) has been displaced from the first line of treatment for most of the HIV-treatment guidelines because of side effects. The prolonged use of these drugs has been convincingly associated with alterations in body fat distribution and metabolic derangements, such as insulin resistance or hypertriglyceridaemia. In naive patients, comparing stavudine to tenofovir (TDF) (plus lamivudine and efavirenz in both arms), stavudine led to significantly greater increases in TG and TC at 96 weeks than TDF [33]. Also, in the 934 study, AZT led to significant greater increases in TC than TDF at 96 weeks, without significant differences in LDL, HDL or TG values [34]. Also, when t-NRTI are switched to TDF or abacavir, TC and TG values are improved [35]. However, we could not find a significant association of t-NRTI on TC/HDL ratio; it is still only speculation that women could in part be protected from lipid derangements associated with the use of t-NRTI.

Our study has several limitations: no information is available regarding HCV viral load or specific HCV treatment. The selection of patients without lipid lowering treatment may bias our study population to a lipid-disturbances protected group of patients. We considered that the inclusion of patients with lipid lowering treatment while on ART adds confusion when interpreting lipid profile. Pharmacologic interactions between statins or fibrates with ARV drugs and different dosages of drugs may render heterogeneous results on lipids that are difficult to analyse. Absence of detailed information about specific drugs is also a limitation of the study. On the other hand, it has the strength to have a high number of patients studied.

## Conclusions

In summary, we have shown that in HIV infected women, NNRTI-based ART is associated with a better lipid profile than the PI-based. The beneficial effect on lipids of NNRTI should make their use advisable in patients with increased cardiovascular risk, such as older women. Age and TG levels are independent factors significantly associated with increased TC/HDL ratio and HCV coinfection is independently associated with a reduced TC/HDL ratio. These factors must be taken into account when analysing the effect on lipid profile of different types of ART.

## Competing interests

The authors declare that they have no competing interests.

## Authors' contributions

VE and ISL had the original idea. VE wrote the article. MFF did the statistical analysis. All authors contributed in the recruitment, reviewed the final document and made suggestions. All authors read and approved the final manuscript.

## Pre-publication history

The pre-publication history for this paper can be accessed here:

http://www.biomedcentral.com/1472-6874/11/36/prepub
